# Persistent Mullerian Duct Syndrome with Polysplenia and Short Pancreas: A Case Report

**DOI:** 10.31729/jnma.4298

**Published:** 2019-04-30

**Authors:** Umesh Kumar Sharma, Dinesh Kumar Thapa, Dinesh Pokhrel, Amit Kumar Shah

**Affiliations:** 1 Department of Radiology, B&C Medical College Teaching Hospital, Birtamod, Jhapa, Nepal; 2 Department of Neurosurgery, B&C Medical College Teaching Hospital, Birtamod, Jhapa, Nepal; 3 Department of Urology, B&C Medical College Teaching Hospital, Birtamod, Jhapa, Nepal

**Keywords:** *cryptorchidism*, *persistent mullerian duct syndrome*, *short pancreas*

## Abstract

Persistent Mullerian duct syndrome is a rare entity and usually presents with common symptoms of undescended testis and hernia. The syndrome is caused by an insufficient amount of Mullerian inhibiting substance or due to the insensitivity of the target organ to Mullerian inhibiting substance. Polysplenia is a rare congenital disease manifested by multiple small accessory spleens. The association of these two entities, Persistent Mullerian duct syndrome and polysplenia, is rare and has not been reported in the literature. We reported a case of a 27 years old male presented with complains of right flank pain and nausea. Ultrasound showed right ureteric calculus with hydronephrosis and elongated soft tissue mass posterior to bladder. Contrast enhanced Computed Tomography showed soft tissue suggestive of infantile uterine structure with multiple splenculi and short pancreas. He was diagnosed as Persistent Mullerian duct syndrome with unilateral cryptorchidism, polysplenia and short pancreas, coincidentally detected while evaluating for ureteric colic. He underwent Ureteroscopic Lithotripsy with stenting for ureteric calculus, however, he refused laparotomy with excision of mullerian structures.

## INTRODUCTION

Persistent Mullerian Duct syndrome (PMDS) is a rare disorder of sex development characterized by the persistence of Mullerian derivatives, the uterus and/or fallopian tubes, in otherwise normally virilized boys.^1–3^ Despite the normal male genotype (46 XY) and the subsequent normal development of fetal testes, müllerian structures do not regress, either due to the absence of Müllerian Inhibiting Substance (MIS) or lack of response to it.^1,2,4,5^

Polysplenia is manifested by multiple small accessory spleens, rather than a single, full sized, normal spleen. Polysplenia, sometimes occurs, alone, but it is often accompanied by other developmental abnormalities. Conditions associated with polysplenia include GI abnormalities, such as intestinal malrotation, or biliary atresia, short pancreas, also cardiac abnormalities.^6,7^

We report a rare case of PMDS, associated with polysplenia, which has never been reported in the literature.

## CASE REPORT

**Figure 1A,B:**
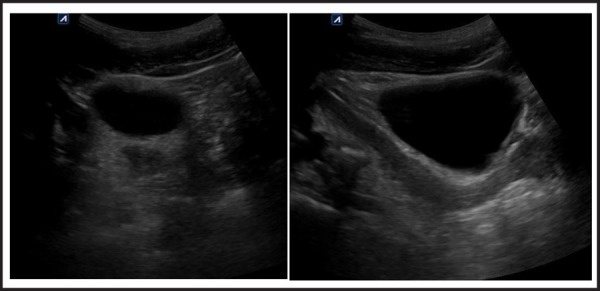
Ultrasound of pelvis: Axial and sagittal sections show uterine structure posterior to urinary bladder and cervix ends at the prostate.

A 27 year old male was presented to the emergency room with acute right flank pain and nausea. The patient is married and has two children. On examination, he was a healthy male with well developed scrotum. Single testis was palpated and there was right sided undescended testis. His urethra and penis were fully developed. Palpation of inguinal areas was unremarkable. The patient was evaluated by ultrasound which showed moderate right hydroureteronephrosis with ureteric calculus. Incidental note of an elongated soft tissue mass posterior to the bladder was made on ultrasound. Left kidney and urinary bladder were unremarkable (Figure 1A,B).

**Figure 2A,B:**
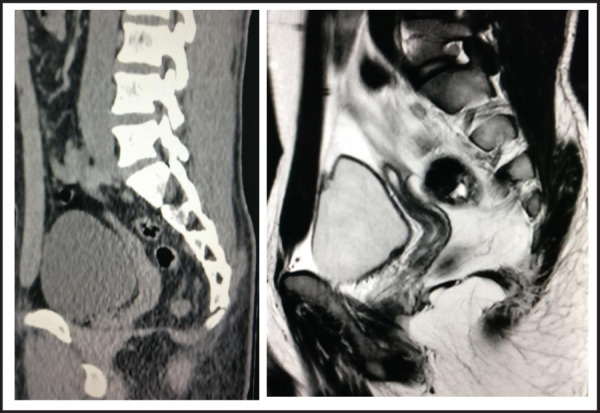
Sagittal image CT scan and MRI T2 WI: Uterus is seen posterior to bladder. The uterus shows hyperintense fluid structure in the endometrial cavity and hypointense junctional line.

**Figure 3A,B:**
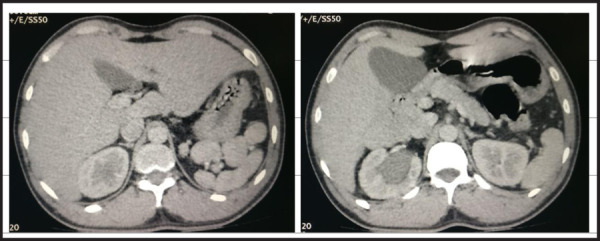
Axial CT scans show multiple accessory spleens and short pancreas. Hydronephrosis in right kidney.

Further evaluation with contrast enhanced CT scan was performed. It showed right ureteric calculus and hydroureteronephrosis. There was a well defined soft tissue density mass measuring 7.8x3.0x2.1cm posterior to the bladder suspicious of an infantile uterine structure. The cervix was abutting the normal sized prostate (Figure 2A). In addition, other incidental findings were multiple rounded splenculi of variable sizes at least 9 in number. The pancreas was short with absence of distal body and tail (Figure 3A and 3B). MRI of pelvis showed similar findings as seen in the CT scan and ultrasound. Sagittal MRI at the level of urinary bladder demonstrate the uterus at midline posterior to bladder. The uterus shows hyperintense fluid signals within the endometrial cavity surrounded by hypointense junctional zone. The lower segment of the uterus extends towards the prostate (Figure 2B). The ovaries were not seen in the pelvis on MRI. The undescended testis could not be found in the pelvis and retroperitoneum in MRI.

Karyotyping was not done in the patient due to unavailability of the facility at the center. The patient underwent URSL with DJ stenting for right ureteric calculus. The patient was advised for the laparotomy and excision of the Mullerian structure, but refused to undergo second surgery.

## DISCUSSION

PMDS is a very rare condition with less than 300 cases described in the literature.^8^ This rare entity portrays adolescents who are phenotypically males with 46, XY karyotypes. Such individuals contain internal female reproductive organs such as Mullerian derivatives, caused by a defect in genes. Mullerian and Wolffian ducts are present in human fetus which differentiates in a male fetus by the end of seven weeks of gestation. The genetic defects that occurred were either in genetic coding for the MIS/anti-Mullerian Hormone (AMH) or the AMH receptor, ultimately leading to failure of regression of Mullerian ducts.^8,9^

Male type and female type are two anatomic variants of PMDS that have been reported. The male form of PMDS is common, encountered in 80–90% of cases and characterized by unilateral cryptorchidism with a contralateral inguinal hernia. The male form of PMDS can be of two types. The first type is hernia uteri inguinalis, which is usually characterized by a descended testis and herniation of the ipsilateral corner of the uterus and the ipsilateral fallopian tube into the inguinal canal.^9,10^ The second type is crossed testicular ectopia, which is characterized by herniation of both testes and the entire uterus and both fallopian tubes.^9,10^

The second anatomic variant of PMDS, the female form, is seen in only 10–20% of cases. It is characterized by bilateral cryptorchidism, with the testes fixed within the round ligaments in an ‘ovarian position’ with respect to the uterus. The gonads are fixed within the pelvis.^9,10^ The mobility of Mullerian structures is an important factor that determines the clinical presentation. If the uterus and fallopian tubes are mobile, they may descend into the inguinal canal during testicular descent. On the other hand, if the Mullerian structures are relatively immobile, testicular descent may be impeded.^2,9,10^

Early diagnosis and surgical treatment is important to prevent sexual-maturation hypertrophy of uterus leading abdominal discomfort and mass secondary to accumulation of blood. The risk of malignancy is nearly 15% in an ectopic testis in a case of PMDS, similar to that in a healthy male.^11^ Chamarajan et al. reported a case of seminoma with bilateral cryptorchidic male with PMDS. Embryonal carcinoma, yolk sac tumor and teratoma can occur in patients with PMDS.^12^ As these patients usually present with a unilateral disease there is a potential chance of preserving their fertility.

Polysplenia, as part of the heterotaxy syndrome, is a rare embryological disorder with a reported incidence of 1 per 250 000 live births.^6^ The precise aetiology of polysplenia is unknown. Embryonic, genetic and teratogenic components have all been implicated as causative factors in polysplenia.^6^ Although polysplenia syndrome has a wide range of abnormalities, there is no single pathognomic abnormality that characterizes this rare entity. The range of anomalies include multiple spleens of equal volume, visceral heterotaxia, right-sided stomach, a left-sided or large midline liver, malrotation of the intestine, a short pancreas and IVC anomalies.^13^

Polysplenia has been reported mainly in childhood owing to critical anatomic malformations related to cardiac defects or biliary atresia. Symptomatic polysplenia in adults is often caused by abnormal biliary and pancreatic duct drainage, cholecystitis and bowel obstruction.^14^ Polysplenia is usually an incidental finding on abdominal ultrasound or CT performed for other causes. Other infrequently reported associations PSS are tracheo-esophageal fistula, renal anomalies, polydactyly, annular pancreas, choledochal cyst and intestinal atresias.^15^

Association of polysplenia with PMDS is rare entity. This case was detected incidentally, while evaluating for acute ureteric colic. There are reports of various associations in PMDS, frequently undescended testis and hernia. In our case, the patient was phenotypically male with cryptorchidism. He was married with two children. On evaluation for the unilateral cryptorchidism, the testis could not be found in the pelvis and retroperitoneum. The patient do have a uterus, but it is of infantile type which is due to absence of oestrogen stimulation, an indirect sign indicating that there is no female hormonal production. Polysplenia was also incidental with multiple splenculi that is more than nine in number. In addition, short pancreas was noted in CT scan with absence of distal body & tail. Imaging modalities, ultrasound, CT and MRI were equally good in detection of the Mullerian abnormality and polysplenia. The developmental anomaly of these two entities, PMDS and polysplenia, could be synchronous without any linkage with embryological or genetical factors. However, such incidence of association has not been reported in the literature so far.

## Consent

**JNMA Case Report Consent Form** was signed by the patient and the original is attached with the patient's chart.

## Conflict of Interest

**None.**
